# Public attitudes towards personal health data sharing in long-term epidemiological research: a Citizen Science approach in the KORA study

**DOI:** 10.1186/s12889-024-19730-0

**Published:** 2024-08-26

**Authors:** Ina-Maria Rückert-Eheberg, Margit Heier, Markus Simon, Monika Kraus, Annette Peters, Birgit Linkohr

**Affiliations:** 1 Institute of Epidemiology, Helmholtz Zentrum München, German Research Center for Environmental Health, Neuherberg, Munich, Germany; 2 KORA Study Centre, University Hospital of Augsburg, Augsburg, Germany; 3 German Centre for Cardiovascular Research (DZHK e.V.), Munich Heart Alliance, Munich, Germany; 4 Institute for Medical Information Processing, Biometry and Epidemiology (IBE), Faculty of Medicine, Ludwig-Maximilians-Universität München, Munich, Germany; 5 Partner Site München-Neuherberg, German Center for Diabetes Research (DZD), Munich-Neuherberg, Germany

**Keywords:** Citizen science, Participatory research, Public engagement, Health data sharing, Epidemiological cohort management, Co-creation

## Abstract

**Background:**

Loss to follow-up in long-term epidemiological studies is well-known and often substantial. Consequently, there is a risk of bias to the results. The motivation to take part in an epidemiological study can change over time, but the ways to minimize loss to follow-up are not well studied. The Citizen Science approach offers researchers to engage in direct discussions with study participants and to integrate their opinions and requirements into cohort management.

**Methods:**

Guided group discussions were conducted with study participants from the KORA cohort in the Augsburg Region in Germany, established 40 years ago, as well as a group of independently selected citizens. The aim was to look at the relevant aspects of health studies with a focus on long-term participation. A two-sided questionnaire was developed subsequently in a co-creation process and presented to 500 KORA participants and 2,400 employees of the research facility Helmholtz Munich.

**Results:**

The discussions revealed that altruistic motivations, (i.e. supporting research and public health), personal benefits (i.e. a health check-up during a study examination), data protection, and information about research results in layman’s terms were crucial to ensure interest and long-term study participation. The results of the questionnaire confirmed these aspects and showed that exclusively digital information channels may be an obstacle for older and less educated people. Thus, paper-based media such as newsletters are still important.

**Conclusions:**

The findings shed light on cohort management and long-term engagement with study participants. A long-term health study needs to benefit public and individual health; the institution needs to be trustworthy; and the results and their impact need to be disseminated in widely understandable terms and by the right means of communication back to the participants.

**Supplementary Information:**

The online version contains supplementary material available at 10.1186/s12889-024-19730-0.

## Background

In a long-term prospective cohort study, the motivation of people to participate over an extended period and trustfully share their health data is essential to investigating causal relationships between health and disease in constantly changing environments. However, loss-to-follow up, i.e. declining willingness to take part in follow-up examinations and questionnaires, is a major problem in all long-term prospective cohort studies [1–3], raising questions about the generalizability of results [4]. Information on the reasons to participate is often gathered at the initial sign-up of the study by short non-participant questionnaires [5–7], satisfaction polls after the study examination for internal conduct improvement, witness statements [8] or by chance when study participants comment to staff or leave remarks in questionnaires. Non-participants often report acute health problems or stressful life-events, but also unspecific reasons like lack of interest or time constraints. In good epidemiological practice, efforts to characterize the loss of follow-up during analysis are made [9] and particular groups can be identified, e.g. less educated groups or middle-aged men, depending on the cohort [10, 11]. However, cohort management should seek to maximize participation in follow-up studies in the first place by trying to meet participants’ expectations. Personal attitudes towards data sharing may change during long-term studies, particularly in the light of the experience of the COVID-19 pandemic. To our knowledge, systematic research into cohort management strategies in long-term epidemiological studies is rare.

Citizen Science, also called “participatory research,” has increasingly been supported by public organizations in and outside of academic institutions to meet information requirements, increase transparency, and improve people’s attitudes towards science [12]. In 2022, the White Paper “Citizen Science Strategy 2030 for Germany” was published that comprehensively informs about Citizen Science, action areas, networking, funding, volunteer management, and many other aspects [13]. Meanwhile, a wide range of scientific projects covering all areas of interest are offered to the public [14–16]. Participatory research strategies have been introduced into health research in various initiatives (e.g. [17]) with the overarching goal “to reduce concerns about the use of data through intensive exchange with interested citizens and to demonstrate the opportunities it offers” [18]. Citizen Science in public health can be characterized by typology according to aim, approach, and size, depending on the level of engagement with the community [19].

Recently, Marcs et al. published a scoping review on Citizen Science approaches in chronic disease prevention where they used Citizen Science to identify problems from the perspective of community members, generate and prioritize solutions, develop, test and/or evaluate interventions, and/or build community capacity [20]. Frameworks for a systematic development of participatory epidemiology have also been proposed [21].

Our aim was to employ Citizen Science approaches to engage in direct discussion with study participants from a well-established epidemiological study to evaluate how to maximise study participation long-term by high response rates and low subsequent withdrawal of consent. We were particularly interested in the reasons for continuing to take part in follow-up studies as well as concerns and wishes regarding the collection and use of health data. The research methods combined Citizen Science approaches like qualitative research and co-design elements with a classical quantitative approach in a nested but work-efficient study design. The project was conducted in a randomly selected subgroup of participants of a long-term prospective cohort study and, for comparison, a group of independent citizens and employees of a large health research institution.

## Methods

The Citizens Science project was embedded in the KORA study (Cooperative Health Research in the Region of Augsburg), an adult population-based prospective cohort study established in 1984 in the City of Augsburg and the adjacent rural counties Augsburg and Aichach-Friedberg in Southern Germany [22]. Briefly, the KORA-study consists of four cross-sectional baseline surveys (S1 from 1984/85 with *N* = 4,022 (response: 79.3%); S2 from 1989/90 with *N* = 4,940 (response: 76.9%); S3 from 1994/95 with 4,856 (response: 74.9%); and S4 from 1999/2001 with 4,261 (response: 66.8%)). The participants were randomly selected from population registries aged 25–74 years (S1: 25–64 years). The KORA study is still in active follow-up with a KORA study centre located in the City of Augsburg. A general health survey was sent out in 2021 to all S1 to S4 participants still living in the study area and with consent for recontact. 6,070 out of 9,109 participants answered the survey (66.6%).

The starting point of the project was qualitative research with three guided discussion groups: two with KORA study participants and one with newly recruited citizens. In a co-creation process at a subsequent meeting, a questionnaire was developed with a smaller group of volunteers from the discussion groups. For the quantitative part of the study, this questionnaire was mailed to participants of the KORA study and distributed to all employees of Helmholtz Munich.

### Discussion groups

During the preparation of the study setup, a pilot discussion group was conducted with seven acquaintances of the involved scientists. For the two discussion groups with KORA volunteers, 183 KORA study participants were selected (criteria: 50% women, 50% participants of the latest KORA general health survey 2021 with online survey completion and 50% paper-based completion, born 1949–1969, residing in Augsburg or nearby). They were invited in writing by post and contacted by telephone. Citizens were recruited via a newsletter advertisement of the Volunteer Centre Augsburg [23], and posters and flyers that were distributed in shops, restaurants, the library, the University Hospital Augsburg, and other public places in Augsburg. To compensate expenses, e.g. for travelling, we paid a small expense allowance.

The discussions took place between May and June 2023 in the KORA Study Centre in Augsburg. Following a short impulse presentation on the KORA study, the attendees were asked to note their motivations, concerns, and wishes regarding the participation in a long-term observational health study separately on index cards. The number of cards was not specified. The participants had the opportunity to present each card to the group before it was displayed on a whiteboard sorted by the respective category. Guided by two moderators, the raised aspects were discussed in greater depth along with a set of prepared questions. To provide more information on data privacy and protection in the KORA study, the consent form and study information from the most recent KORA general health survey in 2021 were distributed. Each discussion group lasted about 90 min and was rounded up with a little get-together at the end. The discussions were audiotaped with Audacity^®^ 3.2.5 and a microphone of the conference system Logitech CC3000e ConferenceCam and transcribed subsequently. In the aftermath, the index cards were coded according to reoccurring themes. One of the authors, who was part of all three discussion groups, developed a coding scheme with the help of the audiotapes. The scheme was reviewed by another author who was not present at the discussions, and consensus was found in terms of discrepant interpretation. Anonymized quotes were selected and translated for publication purposes.

### Questionnaire development

The discussion group participants were invited to a subsequent meeting to develop the questionnaire together with the researchers in a co-creation process. The aim was to recruit six volunteers (two per group) to discuss a prepared questionnaire draft in the light of the results from the discussion groups. The questionnaire was designed for mailing to the KORA study participants first and modified slightly for the employees of Helmholtz Munich thereafter. It consisted of questions on the three pre-defined categories motivations, concerns, and wishes and a section on personal data such as sex, age, and school education. Many of the questions were formatted as 5-point Likert scales.

The questionnaire was piloted at the Institute of Epidemiology, and the final version was also translated into English for the Helmholtz Munich employees (Supplement).

### Questionnaire survey

The paper version was posted to 500 selected KORA participants, equally balanced by sex. They were randomly chosen from the KORA S1-S3 studies from a total of *N* = 2,933 participants born between 1964 and 1945, still living in the study area, and with consent for recontact. 400 of them had taken part in the latest KORA general health survey in 2021, while 100 had not. The approximately 2,400 Helmholtz employees were invited to complete the questionnaire personally on paper in the canteen on campus or online (in PDF format).

### Ethics approval and consent to participate

All discussion group participants gave their written informed consent to take part in the discussions. The questionnaire was conducted anonymously, and no written informed consent was required. This study protocol was approved by the ethics committee of the Bavarian Medical Association (EC 23010).

### Statistical analysis

**Fig. 1:**
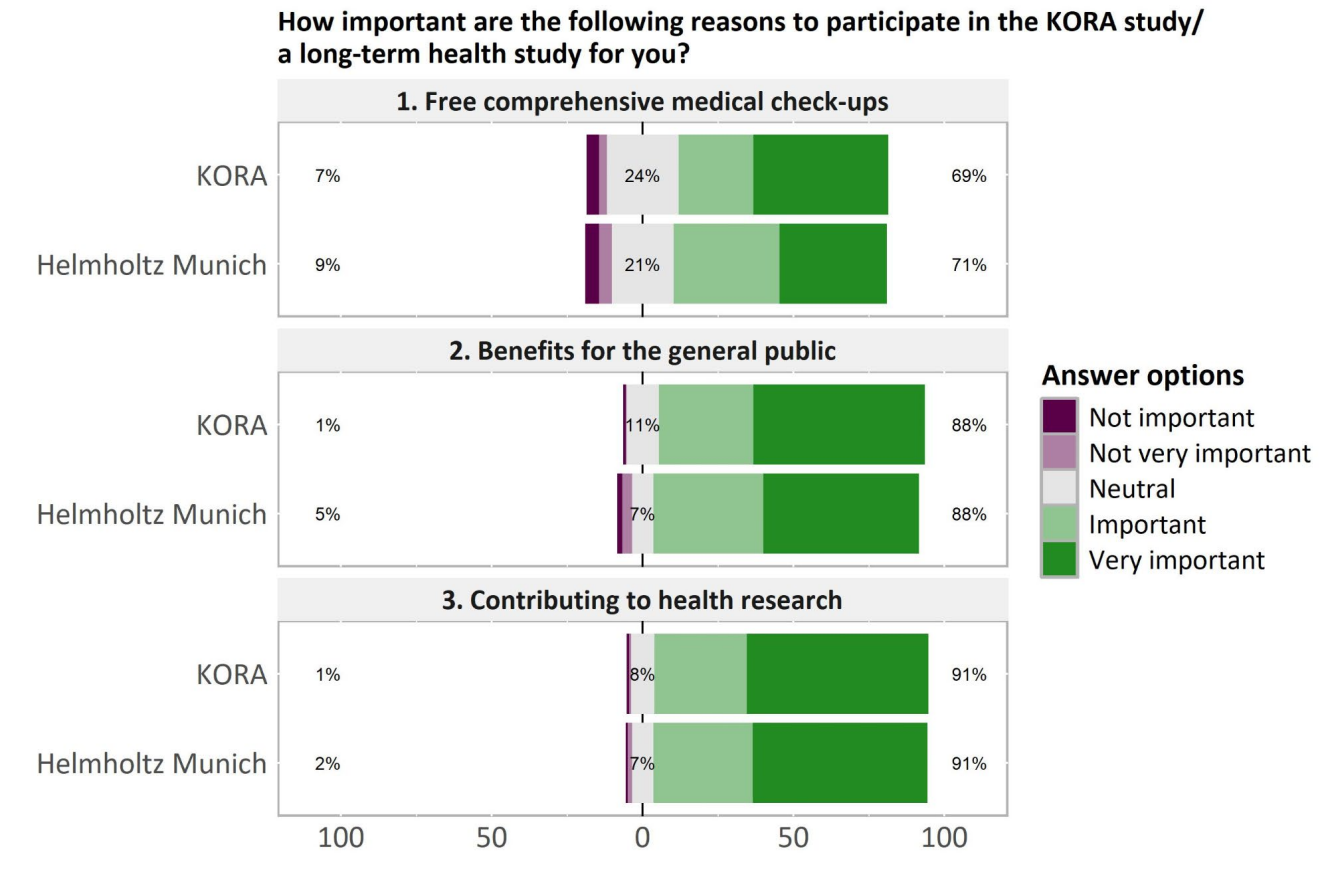
Reasons to participate in the KORA study or a long-term health study. Percentages on the left represent purple responses, percentages on the right represent green responses

**Fig. 2:**
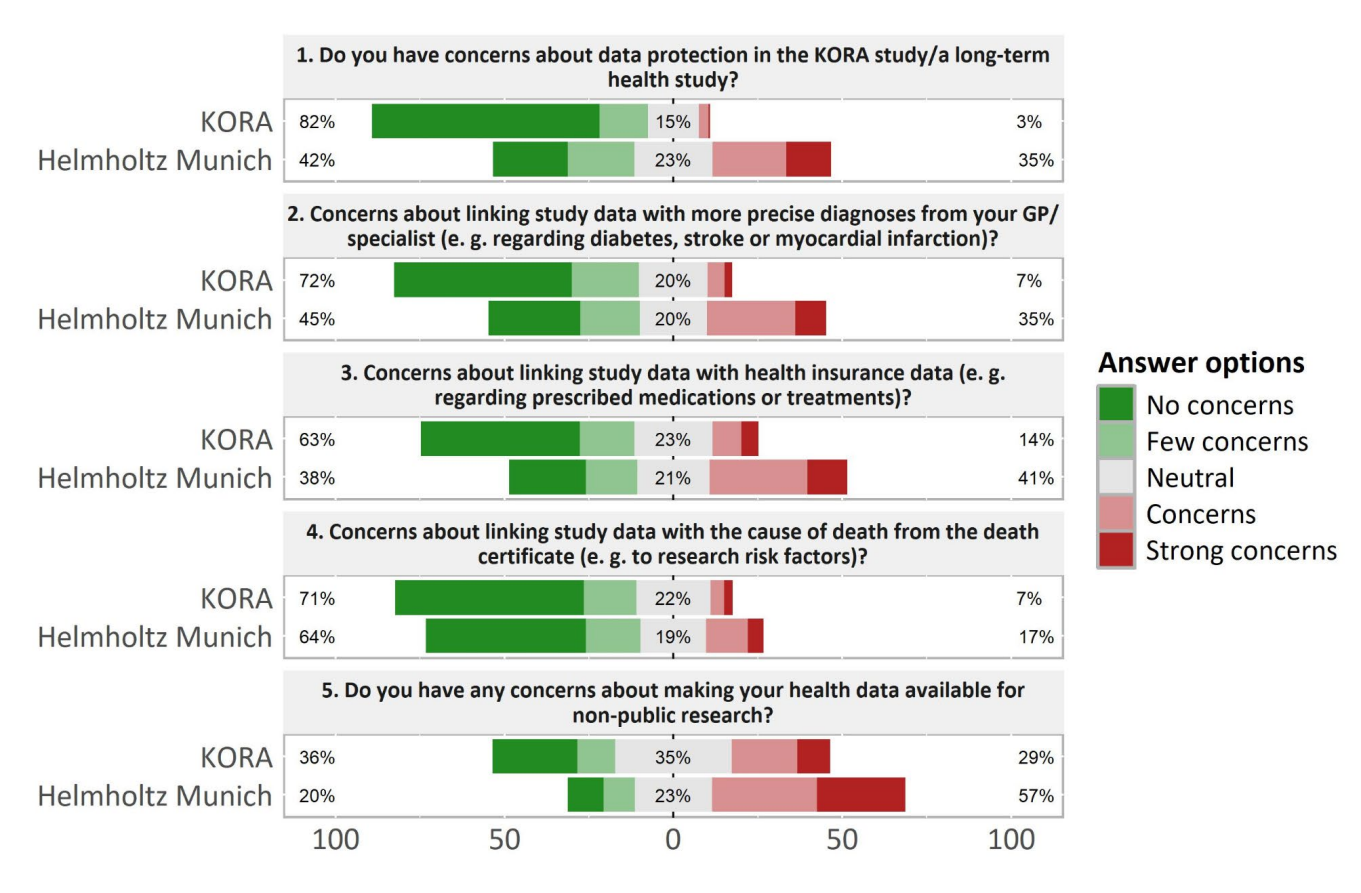
Concerns about data protection, linkage of study data with secondary health information, and use for non-public research. Percentages on the left represent green responses, percentages on the right represent red responses

**Fig. 3:**
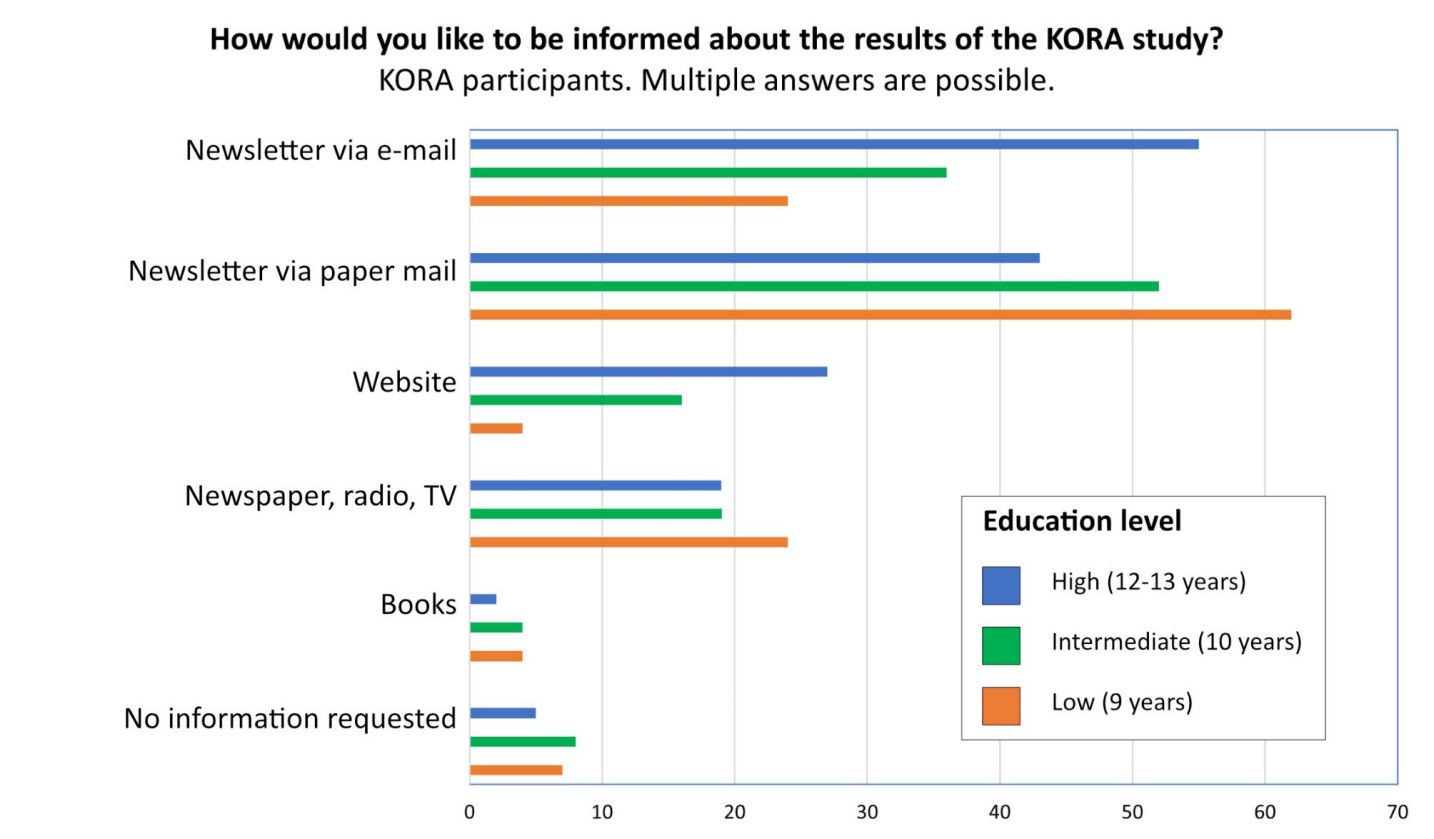
Preferred information channels to disseminate research results of the KORA study, stratified by school education

The data from the completed questionnaires was transferred to a database and analyzed primarily with R and RStudio (Boston, MA, USA). Characteristics of the qualitative study groups were reported with absolute numbers, and characteristics of the quantitative questionnaire study population with numbers and percentages. The R-package „Likert“ was used to create Likert scale charts (Figs. 1 and 2). Percentages were calculated to sum the two categories “not important” and “not very important”, and the two categories “important” and “very important”, respectively. The category “neutral” was also visualized, and the percentages were given. Figure 3 was set up in Excel. Percentages were calculated and displayed by education level after exclusion of participants with missing information on education (*N* = 1) and those who had no school-leaving certificate (*N* = 2). Significance tests were not performed because the statistics were descriptive and not adjusted for confounding factors.

## Results

### Discussion groups

Twenty-four people participated in the three discussion groups (17 probands of the KORA study, 7 citizens, 11 women, and 13 men). Their age range was 42 to 78 years (mean age: 65 years). 14 people reported high (12–13 years), 9 intermediate (10 years), and one person low (9 years) school education.

Table 1 shows the results of the group discussions stratified by category. There was no major difference between KORA participants and the citizen group. Most ideas were raised in the category motivations, followed by wishes and concerns. We excluded statements that went beyond the scope of a health study (concerns: general criticism of the health system (3x) and study staff would not listen (1x); wishes: individual health advice (8x) and contact between participants (2x)).

The number of people who referred to one of the aspects listed in the table is depicted in column N.

**Table 1:** Discussion group results on motivations, concerns, and wishes

Motivations (*N* = 38)		Concerns (*N* = 21)		Wishes (*N* = 26)	
*N*	Label	*N*	Label	*N*	Label
14	Free preventive medical check-up	10	Data protection and data security	9	Communication of the study results to the participants
11	Benefits for the public	2	Incorrect use of the results	7	More frequent examinations/additional studies
7	Contribution to research	2	Right not to know	4	Simple, brief, and comprehensible communication
3	Interest in health research	1	Use in industry	3	Information about how research findings are used to the healthcare system
3	Professional study organization	2	No concern	2	Incentives, financial or non-financial

For many volunteers, a motivation to take part in the KORA study or a health study in general was the free preventive medical check-up in the form of the study examinations.

*Discussion Group 1*,* KORA participant: “So*,* my motivation to join was to get information about my health that I wouldn’t have gotten otherwise.”*

Additionally, the discussants placed great importance on the benefits for the public, their contribution to health research, and their interest in it.

*Discussion Group 3*,* KORA participant: “In terms of motivation*,* the focus is*,* of course*,* quite clearly on the fact that the benefit is for the general public.”*

*Discussion Group 1*,* KORA participant: “And then*,* of course*,* that one contributes to general research.”*

The professional conduct of the study was also mentioned several times.

The participants raised fewer issues in the category concerns than in the categories motivations and wishes. The main aspects were protection and security of health data in KORA or generally in health studies.

*Discussion Group 1*,* KORA participant: “My concerns are (…) data protection and data usage. Not particularly in relation to Helmholtz Munich*,* but the overall (…) misuse*,* data hackers*,* cybercrime*,* all that stuff. And that will increase even more in the future.”*

*Discussion Group 2*,* Citizen: “…it is always difficult with data protection in an international comparison. We have very high standards here*,* but can we maintain them in the long term? Because*,* of course*,* we also create barriers that are incomprehensible to others.”*

Some of these concerns were not directed at the discussants themselves but rather at younger people who might suffer greater harm through misuse. Discrimination in professional life or when taking out insurance were mentioned as examples in this context.

*Discussion Group 1*,* KORA participant: “Personally*,* I wouldn’t mind (…)*,* but with younger*,* working people*,* I would probably have a different opinion. Because today*,* you can supposedly already say that people might get certain diseases at some point. (…) And I think that is dangerous if this information goes to the insurance companies or to the employers themselves (…).”*

The participants did express their trust in Helmholtz Munich as a publicly funded research institution, and the consent form and study information were considered informative and clear; some participants even found them too detailed.

A minority of the participants had no worries whatsoever.

*Discussion Group 3*,* KORA participant: “I really can’t say anything about concerns. If my data were published with my name*,* I wouldn’t care at all.”*

In the category wishes, the participants pointed out that more communication on study results and their translation into the health care system would motivate them long-term to participate in a study.

*Discussion Group 2*,* Citizen: “(…) the research results must be disseminated more widely. In my opinion*,* they have primarily been intended for experts.”*

*Discussion Group 2*,* Citizen: “I find the contributions on the Internet (…) terrible. The layperson gets all mixed up. You’d have to clean up that mess*,* too.”*

Many participants indicated that simple, brief, and comprehensible communication was appreciated. Some discussants preferred digital formats, while others explicitly stated that they wanted paper-based communication only. Overall, the discussion group participants were open to health research and were interested in more frequent examinations and additional study offers.

### Questionnaire development

A two-page questionnaire was developed in a meeting between two out of the 24 discussion group participants and two researchers. The participants pointed out some complicated questions and assessed the overall comprehensibility.

### Questionnaire survey

The survey was completed by 278 KORA participants (response rate: 67% in those who had participated in the latest KORA follow-up and 9% in those who had not participated) and 285 Helmholtz Munich employees (response rate: about 12% as the exact number of employees was not available), resulting in a total study population of 563 people. The characteristics of the study population are displayed in Table 2. Approximately the same number of women and men took part in the survey. The KORA study participants were between 58 and 78 years old (mean age: 67.9 years). The Helmholtz Munich employees were younger, mostly between 20 and 50 years old (mean age: 39.8 years). About one-third of the KORA participants had low (9 years), intermediate (10 years), and high (12–13 years) levels of school education. In contrast, most of the Helmholtz Munich employees (89.2%) had a high level of education. 71.4% of the Helmholtz Munich employees worked scientifically, and 70.4% had German citizenship.

**Table 2:** Characteristics of the questionnaire survey population

	KORA (*N* = 278)	Helmholtz Munich (*N* = 285)
**Sex**		
Men	141 (50.9%)	131 (46.1%)
Women	136 (49.1%)	152 (53.5%)
Diverse	0 (0%)	1 (0.4%)
Missing	1 (0.4%)	1 (0.4%)
**Age**		
20–29 years	0 (0%)	73 (25.8%)
30–39 years	0 (0%)	80 (28.3%)
40–49 years	0 (0%)	61 (21.6%)
50–59 years	26 (9.4%)	51 (18.0%)
60–69 years	130 (46.8%)	17 (6.0%)
70–78 years	122 (43.9%)	1 (0.4%)
Missing	0 (0%)	2 (0.7%)
**School education**		
Low	103 (37.2%)	9 (3.2%)
Intermediate	83 (30.0%)	20 (7.2%)
High	89 (32.1%)	247 (89.2%)
No school leaving certificate	2 (0.7%)	1 (0.4%)
Missing	1 (0.4%)	8 (2.8%)
**Focus of work at Helmholtz Munich**		
Science	NA	202 (71.4%)
Administration/infrastructure	NA	81 (28.6%)
Missing	NA	2 (0.7%)
**German citizenship**		
Yes	NA	200 (70.4%)
No	NA	84 (29.6%)
Missing	NA	1 (0.4%)

In the questionnaire, participants were asked how important they rated the three listed reasons to participate in the KORA study or a long-term health study (Fig. 1). The answers of the KORA study participants and the Helmholtz employees were very similar. A majority of about 90% deemed “contributing to health research” and “benefits for the general public” as very important or important. “Free comprehensive medical check-ups” were also seen as important or very important by about 70%, while about 20% took a neutral position on this aspect.

Differences between the two participant groups were found regarding questions about concerns in relation to data protection and data linkage (Fig. 2). Only a small proportion of the KORA study participants had reservations about data protection in the KORA study (3%). Concerns or strong concerns increased with regards to linking their study data to secondary health data such as diagnoses by their physicians (7%), prescription and treatment data by their health insurance (14%), but it decreased with regards to the cause of death sometime in the future (7%). In comparison, 35% of the Helmholtz Munich employees had concerns or strong concerns about data protection in a long-term health study. Data linkage was seen critically by 35% regarding study and physician diagnosis data, by 41% regarding study and health insurance data, and by 17% regarding study and death certificate data.

A larger proportion in both groups (29% of the KORA participants and 57% of the Helmholtz Munich employees) indicated concerns or strong concerns about the utilization of their health data by non-public research organizations.

The KORA participants were asked how they would like to be informed about the research results of the KORA study. Multiple selections were allowed. Figure 3 shows the percentages stratified by school education. Participants with a high level of school education preferred digital channels such as electronic newsletters and websites, in contrast to participants with low or intermediate school education, who preferred information, i.e. newsletters by paper mail. About 20% of each group indicated that they would appreciate coverage of scientific research results via newspapers, radio, and TV, while books were only interesting for a small proportion of participants. Less than 10% did not wish for any information. Of the 147 participants who chose a newsletter by paper mail, 20% also selected a newsletter by email, and 4% also selected the website category – thus, 77% of those who chose paper mail wanted no digital information.

## Discussion

Using Citizen Science approaches, this project examined the motivations, concerns, and wishes of research participants to help slow down the decline in follow-up study participation. The KORA study was established almost four decades ago and is still in active follow-up with relatively high response rates, e.g. 64% in an examination in 2018/19 [24] and 66.7% in a general health survey in 2021. Longitudinal data is particularly informative for life-course health research, but few studies exist on how to keep up motivation in follow-up studies. The findings from the discussion groups and the questionnaire survey showed that participants can be motivated to provide their personal health data for scientific purposes over long periods of time if their expectations are met. Three main reasons to participate in a long-term health study were identified: the benefit to the public, scientific progress, and personal health. Those findings are consistent with a previous study led by KORA scientists in 2010 on the public perceptions of cohort studies and biobanks during the recruitment phase of the German National Cohort (NAKO) [25]. They found that in general, citizens approve epidemiological research based on expectations for communal and individual benefits (e.g., health check-ups and health information). This shows that the basic motivation for study participation does not change between study initiation and long-term follow-up. Collaboration with science [26], making a contribution to society [27], and receiving information about personal health [28] have also been known as motivations for study participation in clinical studies. In a recent study on retaining participants in longitudinal studies of Alzheimer’s disease, altruism and personal benefit were the factors associated with continued study participation as well [29].

In the discussion groups, data protection did not come up as a major concern and was not necessarily directed at the KORA study. In the questionnaire, participants had no strong concerns about their data in the KORA study, even for data linkage. This is in line with the findings by Bongartz et al. that the trustworthiness of those conducting research appeared to be most important for the decision to participate in a health-related study [30]. However, Helmholtz Munich employees expressed more concerns with regards to data protection and data linkage. A likely interpretation for this difference is that KORA participants referred to a specific study that they had a lot of experience with, while Helmholtz employees imagined some theoretical long-term health study. Moreover, the Helmholtz employees were, on average, younger, higher educated, and probably more informed about data protection and data security risks. Our findings showed that institutional trust is essential for long-term participation in a health study. Once trust is gained at initial sign-up, it is important to maintain it. The comprehensive study by Tommel et al. also supports the importance of trust [31]. They explored citizens and healthcare professionals’ perspectives on personalized genomic medicine and personal health data spaces in questionnaires and interviews. Cohort management can help maintain trust, but overall satisfaction with the health system, public health policy, or pandemics is outside its scope.

About one-third of the KORA participants and about two-thirds of the Helmholtz Munich employees expressed concern about sharing data with non-public research organizations. This is in line with findings that people are generally prepared to participate in epidemiological research if it is conducted by a trusted public institution, but that there is widespread distrust of research conducted or sponsored by pharmaceutical companies [32, 33]. However, this degree of concern in both groups was somewhat surprising, as most KORA participants had given consent to sharing their data with industry previously, and Helmholtz Munich contributes to the translation of research into medical innovation with commercial partners.

The discussion group participants wished to be informed about the results and impact of the research in a generally understandable format. The information should be addressed to them personally, such as through a newsletter, rather than in the press, TV, or the internet. A notable proportion of the KORA participants wished to be informed via non-digital means. This is an important finding for those running population-based studies such as the German National Cohort [34] and their financing bodies. While the finding may be specific to the setting in Southern Germany and a long-term cohort study with aged participants, it is important to monitor the information preferences. In addition to digital tools, paper-based methods are still needed for many more years to not lose large groups of the general population. Future research should focus particularly on the digital readiness of older citizens, so that cohort management strategies can engage participants at their level. In long-term health studies, morbidity and mortality are often relevant health outcomes. Public health policies that enable secondary data linkage could also compensate for loss to follow-up and limit selection bias.

### Strengths and limitations

A strength of this project is its diverse group of participants, which includes stakeholders from a long-term epidemiological study, independent citizens, and staff from a research institution earning their living in health science research.

The discussion groups were structured but allowed participants to explain their own narratives and introduce new issues. The questionnaire was administered to two very different groups of participants, and in part, similar results were obtained that confirmed each other (i.e., important motivations to take part in health research (Fig. 1)).

With respect to limitations, a Citizen Science project depends on participants who are interested and motivated to take part. It is quite difficult to find enough participants, and only 24 discussion group volunteers do not necessarily represent the “general” public, especially as discussants with low education were underrepresented. Participants living in rural areas were completely absent due to the recruitment strategy that they had to live in a reasonable travel distance from the KORA study center. The dates and times of the discussion groups were fixed by the researchers and probably discouraged very busy people. However, the small fee and snacks seemed to motivate some of the participants with lower economic status to take part.

In addition, it cannot be ruled out that the ideas of the discussants as well as the answers of the questionnaire survey were influenced by social desirability, perhaps on a subconscious level, and people might thus act somewhat differently in real life than they indicated they would in a theoretical setting. In a group discussion, participants may give answers that they believe to be expected and that will please the interviewer or moderator. Social desirability bias was certainly less of an issue in the questionnaire survey as it was anonymous. However, the outcomes of the discussion groups generally agreed with the responses to the questionnaire given to KORA participants. This questionnaire represents the views of a pre-selected group of people who were recruited up to forty years ago and who still consent to be contacted again for follow-up research. The response to the questionnaire by the KORA participants was as expected: It was high among those who had participated in the latest KORA general health survey in 2021, but it was very low in those who did not participate at the time. This shows that participants who are lost to follow-up are difficult to re-engage.

Finally, the development of the questionnaire was intended to be a co-creation process between selected discussion group participants and scientists. However, the interest of the discussion group members in co-creation was low, and only two participants were willing to take part in this process. They improved the comprehensibility of the questionnaire draft but saw themselves clearly as contributors rather than co-creators. A successful co-creation process requires more capacity building than was possible in this project. As Laird et al. pointed out, Citizen Science approaches often face barriers like building up longer-term collaborative relationships, and their implementation is often time and resource constrained [35].

## Conclusions

The Citizen Science approach opens a new possibility to get in touch with study participants more closely and to integrate their opinions and requirements into cohort management.

On the one hand, people are altruistically motivated when they decide to take part in a long-term health study, and they enjoy the possibility to contribute to public benefit and scientific progress. On the other hand, they also see benefits for their personal health. Concerns do not seem to prevail. Feedback in layman’s terms on the long-term results of the study is highly appreciated and should be addressed to the participant personally.

Cohort management should include regular feedback of results as a thank you for the data donation and contribution to society.

In other words, a long-term health study needs to benefit public and individual health, to be trustworthy regarding data protection and data use, and to provide long-term research results in generally understandable terms and in the preferred communication mode back to the participants.

### Electronic supplementary material

Below is the link to the electronic supplementary material.

Supplementary Material 1

## Data Availability

The datasets used and/or analyzed during the current study are available from the corresponding author on reasonable request via the application tool KORA.passt (https://helmholtz-muenchen.managed-otrs.com/external/).

## Abbreviations

KORA: Cooperative Health Research in the Region of Augsburg

## References

[1] Osler M, Linneberg A, Glumer C, Jorgensen T. The cohorts at the Research Centre for Prevention and Health, formerly ‘The Glostrup Population studies’. Int J Epidemiol. 2011;40(3):602–10. 20338891 10.1093/ije/dyq041

[2] Rabel M, Meisinger C, Peters A, Holle R, Laxy M. The longitudinal association between change in physical activity, weight, and health-related quality of life: results from the population-based KORA S4/F4/FF4 cohort study. PLoS ONE. 2017;12(9):e0185205. 28953956 10.1371/journal.pone.0185205 PMC5617179

[3] Volzke H, Schossow J, Schmidt CO, Jurgens C, Richter A, Werner A, et al. Cohort Profile Update: the study of Health in Pomerania (SHIP). Int J Epidemiol. 2022;51(6):e372–83. 35348705 10.1093/ije/dyac034

[4] Zivadinovic N, Abrahamsen R, Pesonen M, Wagstaff A, Toren K, Henneberger PK, et al. Loss to 5-year follow-up in the population-based Telemark Study: risk factors and potential for bias. BMJ Open. 2023;13(3):e064311. 36997259 10.1136/bmjopen-2022-064311 PMC10069543

[5] Hoffmann W, Terschüren C, Holle R, Kamtsiuris P, Bergmann M, Kroke A, et al. [The problem of response in epidemiologic studies in Germany (Part II)]. Gesundheitswesen. 2004;66(8–9):482–91. 15372348 10.1055/s-2004-813094

[6] Enzenbach C, Wicklein B, Wirkner K, Loeffler M. Evaluating selection bias in a population-based cohort study with low baseline participation: the LIFE-Adult-study. BMC Med Res Methodol. 2019;19(1):135. 31262266 10.1186/s12874-019-0779-8 PMC6604357

[7] Holle R, Hochadel M, Reitmeir P, Meisinger C, Wichmann HE. Prolonged recruitment efforts in health surveys: effects on response, costs, and potential bias. Epidemiology. 2006;17(6):639–43. 17003684 10.1097/01.ede.0000239731.86975.7f

[8] NaKo - Botschafter. https://nako.de/studie/nako-botschafter/. Accessed 07 May 2024.

[9] Nohr EA, Liew Z. How to investigate and adjust for selection bias in cohort studies. Acta Obstet Gynecol Scand. 2018;97(4):407–16. 29415329 10.1111/aogs.13319

[10] Powers J, Tavener M, Graves A, Loxton D. Loss to follow-up was used to estimate bias in a longitudinal study: a new approach. J Clin Epidemiol. 2015;68(8):870–6. 25700941 10.1016/j.jclinepi.2015.01.010

[11] Kendall CE, Raboud J, Donelle J, Loutfy M, Rourke SB, Kroch A, et al. Lost but not forgotten: a population-based study of mortality and care trajectories among people living with HIV who are lost to follow-up in Ontario, Canada. HIV Med. 2019;20(2):88–98. 30474908 10.1111/hiv.12682 PMC9292000

[12] European Citizen Science Platform. https://eu-citizen.science. Accessed 07 May 2024.

[13] Bonn A, Brink W, Hecker S, Herrmann TM, Liedtke C, Premke-Kraus M, Voigt-Heucke S et al. White Paper Citizen Science Strategy 2030 for Germany (https://www.mitforschen.org/sites/default/files/grid/2024/07/24/White_Paper_Citizen_Science_Strategy_2030_for_Germany.pdf) 2022.

[14] mit:forschen!. Gemeinsam Wissen schaffen (ehemals Bürger schaffen Wissen). www.buergerschaffenwissen.de. Accessed 07 May 2024.

[15] Zooniverse. People-powered research. www.zooniverse.org. Accessed 07 May 2024.

[16] European Commission - Marie. Skłodowska-Curie Actions. https://marie-sklodowska-curie-actions.ec.europa.eu/news/marie-sklodowska-curie-actions-funds-44-projects-to-bring-research-closer-to-education-and-society-across-europe. Accessed 08 May 2024.

[17] Schütt AM-F, Weschke E. Sarah. Aktive Beteiligung Von Patientinnen Und Patienten in Der Gesundheitsforschung. Eine Heranführung für (klinisch) Forschende. Bonn/Berlin: DLR Projektträger; 2023.

[18] NFDI4Health. Nationale Forschungsdateninfrastruktur für personenbezogene Gesundheitsdaten. https://www.nfdi4health.de. Accessed 07 May 2024.

[19] Den Broeder L, Devilee J, Van Oers H, Schuit AJ, Wagemakers A. Citizen Science for public health. Health Promot Int. 2018;33(3):505–14. 28011657 10.1093/heapro/daw086 PMC6005099

[20] Marks L, Laird Y, Trevena H, Smith BJ, Rowbotham S. A scoping review of Citizen Science Approaches in Chronic Disease Prevention. Front Public Health. 2022;10:743348. 35615030 10.3389/fpubh.2022.743348 PMC9125037

[21] Bach M, Jordan S, Hartung S, Santos-Hovener C, Wright MT. Participatory epidemiology: the contribution of participatory research to epidemiology. Emerg Themes Epidemiol. 2017;14:2. 28203262 10.1186/s12982-017-0056-4 PMC5301332

[22] Holle R, Happich M, Lowel H, Wichmann HE, Group MKS. KORA–a research platform for population based health research. Gesundheitswesen. 2005;67(Suppl 1):S19–25. 16032513 10.1055/s-2005-858235

[23] Freiwilligen-Zentrum Augsburg. https://www.freiwilligen-zentrum-augsburg.de/. Accessed 07 May 2024.

[24] Rooney JP, Rakete S, Heier M, Linkohr B, Schwettmann L, Peters A. Blood lead levels in 2018/2019 compared to 1987/1988 in the German population-based KORA study. Environ Res. 2022;215(Pt 1):114184. 36041540 10.1016/j.envres.2022.114184

[25] Starkbaum J, Gottweis H, Gottweis U, Kleiser C, Linseisen J, Meisinger C, et al. Public perceptions of cohort studies and biobanks in Germany. Biopreserv Biobank. 2014;12(2):121–30. 24749879 10.1089/bio.2013.0071

[26] Costas L, Bayas JM, Serrano B, Lafuente S, Muñoz MA. Motivations for participating in a clinical trial on an avian influenza vaccine. Trials. 2012;13:28. 22452976 10.1186/1745-6215-13-28 PMC3348022

[27] Richter G, Krawczak M, Lieb W, Wolff L, Schreiber S, Buyx A. Broad consent for health care-embedded biobanking: understanding and reasons to donate in a large patient sample. Genet Med. 2018;20(1):76–82. 28640237 10.1038/gim.2017.82

[28] Akmatov MK, Jentsch L, Riese P, May M, Ahmed MW, Werner D, et al. Motivations for (non)participation in population-based health studies among the elderly - comparison of participants and nonparticipants of a prospective study on influenza vaccination. BMC Med Res Methodol. 2017;17(1):18. 28148221 10.1186/s12874-017-0302-z PMC5288977

[29] Gabel M, Bollinger RM, Coble DW, Grill JD, Edwards DF, Lingler JH, et al. Retaining participants in Longitudinal studies of Alzheimer’s Disease. J Alzheimers Dis. 2022;87(2):945–55. 35404282 10.3233/JAD-215710 PMC9673904

[30] Bongartz H, Rübsamen N, Raupach-Rosin H, Akmatov MK, Mikolajczyk RT. Why do people participate in health-related studies? Int J Public Health. 2017;62(9):1059–62. 28861618 10.1007/s00038-017-1032-z

[31] Tommel J, Kenis D, Lambrechts N, Brohet RM, Swysen J, Mollen L et al. Personal Genomes in Practice: Exploring Citizen and Healthcare Professionals’ Perspectives on Personalized Genomic Medicine and Personal Health Data Spaces Using a Mixed-Methods Design. Genes (Basel). 2023;14(4). 10.3390/genes14040786 PMC10137790 37107544

[32] Slegers C, Zion D, Glass D, Kelsall H, Fritschi L, Brown N, et al. Why do people participate in epidemiological research? J Bioeth Inq. 2015;12(2):227–37. 25672617 10.1007/s11673-015-9611-2

[33] Richter G, Borzikowsky C, Lesch W, Semler SC, Bunnik EM, Buyx A, et al. Secondary research use of personal medical data: attitudes from patient and population surveys in the Netherlands and Germany. Eur J Hum Genet. 2021;29(3):495–502. 33005018 10.1038/s41431-020-00735-3 PMC7940390

[34] Peters A, German National Cohort C, Peters A, Greiser KH, Gottlicher S, Ahrens W, et al. Framework and baseline examination of the German National Cohort (NAKO). Eur J Epidemiol. 2022;37(10):1107–24. 36260190 10.1007/s10654-022-00890-5 PMC9581448

[35] Laird Y, Marks L, Smith BJ, Walker P, Garvey K, Jose K et al. Harnessing citizen science in health promotion: perspectives of policy and practice stakeholders in Australia. Health Promot Int. 2023;38(5). 10.1093/heapro/daad101 PMC10500964 37706963

