# Supplementary material for: Public attitudes towards personal health data sharing in long-term epidemiological research: a Citizen Science approach in the KORA study
Source: BMC Public Health. 2024 Aug 26;24:2317. doi: 10.1186/s12889-024-19730-0 (PMC11348671; doi:10.1186/s12889-024-19730-0)
Supplement: Supplementary file 1 — Supplementary Material 1 [file 12889_2024_19730_MOESM1_ESM.pdf]

MIKA Questionnaire for KORA study participants

A. Motivation

1) How important are the following reasons for you to participate in the KORA study?

	Not important 1	2	Neutral 3	4	Very important 5
Free comprehensive medical check-ups	☐	☐	☐	☐	☐
Benefits for the general public	☐	☐	☐	☐	☐
Contributing to health research	☐	☐	☐	☐	☐

2) Overall, have your expectations of the KORA study been met so far?

☐ 1 Fully ☐ 2 For the most part ☐ 3 Rather less ☐ 4 Not at all

Space for comments:

____

3) What would be important for you to **continue long-term** to participate in the KORA study?

	Not important 1	2	Neutral 3	4	Very important 5
Telephone interview instead of a questionnaire	☐	☐	☐	☐	☐
Short questionnaires	☐	☐	☐	☐	☐
Short examinations	☐	☐	☐	☐	☐
Taxi services to the study centre	☐	☐	☐	☐	☐
Home visits	☐	☐	☐	☐	☐
Involvement of relatives or carers	☐	☐	☐	☐	☐
Financial compensation	☐	☐	☐	☐	☐
Offer of further interesting studies	☐	☐	☐	☐	☐
Professional study conduct	☐	☐	☐	☐	☐

B. Concerns

4) Do you have concerns about data protection in the KORA study?

	No concerns 1	2	Neutral 3	4	Strong concerns 5
Data protection concerns	☐	☐	☐	☐	☐

5) Have your concerns about data protection changed in recent years in relation to the KORA study?

☐ 1 Yes, increased ☐ 2 Yes, decreased ☐ 3 No change

©2023 This work is licensed under Creative Commons Attribution 4.0 International

6) For some research questions, it is necessary to expand your information from the study with information from health care providers. Please rate your concerns by category.

	No concerns 1	2	Neutral 3	4	Strong concerns 5
More precise diagnosis from your GP/ specialist (e.g., on diabetes, stroke, and heart attack)	☐	☐	☐	☐	☐
Cause of death from the death certificate (e.g., to research risk factors)	☐	☐	☐	☐	☐
Data from the health insurance company (e.g., on prescribed medication or treatments carried out)	☐	☐	☐	☐	☐

7) Does the benefit of a health study for the general public outweigh any concerns you may have?

Yes ☐ 1 No ☐ 2

8) Do you have any concerns about making your health data available for non-public research, e.g., to the industry for medical product development?

	No concerns 1	2	Neutral 3	4	Strong concerns 5
Health data for non-public research	☐	☐	☐	☐	☐

C. Wishes

9) How would you like to be informed about research results of the KORA study?

Multiple answers are possible.

☐ 1 Newsletter by e-mail
☐ 2 Newsletter by mail
☐ 3 Website
☐ 4 Newspapers, radio, TV
☐ 5 Non-fiction books
☐ 6 No information wanted

10) Would you like to fill in future questionnaires online? Yes ☐ 1 No ☐ 2

D. Information about my person

11) Age: |__|__| years

12) Gender: Male ☐ 1 Female ☐ 2 Diverse ☐ 3

13) What is your highest general school-leaving qualification?

☐ 1 Secondary school
☐ 2 Secondary school leaving certificate
☐ 3 A-levels
☐ 4 No degree

14) Space for remarks:

©2023 This work is licensed under Creative Commons Attribution 4.0 International

MIKA Questionnaire for Helmholtz Munich employees

A. Motivation

1) How important are the following reasons for you to participate in a **long-term** health study?

	Not important 1	2	Neutral 3	4	Very important 5
Free comprehensive medical check-ups	☐	☐	☐	☐	☐
Benefits for the general public	☐	☐	☐	☐	☐
Contributing to health research	☐	☐	☐	☐	☐

2) What would be important for you to participate in a long-term health study?

	Not important 1	2	Neutral 3	4	Very important 5
Telephone interview instead of a questionnaire	☐	☐	☐	☐	☐
Short questionnaires	☐	☐	☐	☐	☐
Short examinations	☐	☐	☐	☐	☐
Taxi services to the study centre	☐	☐	☐	☐	☐
Home visits	☐	☐	☐	☐	☐
Involvement of relatives or carers	☐	☐	☐	☐	☐
Financial compensation	☐	☐	☐	☐	☐
Offer of further interesting studies	☐	☐	☐	☐	☐
Professional study conduct	☐	☐	☐	☐	☐

B. Concerns

3) Do you have concerns about data protection in a long-term health study?

	No concerns 1	2	Neutral 3	4	Strong concerns 5
Data protection concerns	☐	☐	☐	☐	☐

4) Have your concerns about data protection changed in recent years in relation to a long-term health study?

☐ 1 Yes, increased ☐ 2 Yes, decreased ☐ 3 No change

©2023 This work is licensed under Creative Commons Attribution 4.0 International

5) For some research questions, it is necessary to expand your information from the study with information from health care providers. Please rate your concerns by category.

	No concerns 1	2	Neutral 3	4	Strong concerns 5
More precise diagnosis from your GP/ specialist (e.g., on diabetes, stroke, and heart attack)	☐	☐	☐	☐	☐
Cause of death from the death certificate (e.g., to research risk factors)	☐	☐	☐	☐	☐
Data from the health insurance company (e.g., on prescribed medication or treatments carried out)	☐	☐	☐	☐	☐

6) Does the benefit of a health study for the general public outweigh any concerns you may have?

Yes ☐ 1 No ☐ 2

7) Do you have any concerns about making your health data available for non-public research, e.g., to the industry for medical product development?

	No concerns 1	2	Neutral 3	4	Strong concerns 5
Health data for non-public research	☐	☐	☐	☐	☐

C. Wishes

8) How would you like to be informed about the results of a long-term health study?

Multiple answers are possible.

- ☐ 1 Newsletter by e-mail
- ☐ 2 Newsletter by mail
- ☐ 3 Website
- ☐ 4 Newspapers, radio, TV
- ☐ 5 Non-fiction books
- ☐ 6 No information wanted

D. Information about my person

9) Age: |__|__| years

10) Gender: Male ☐ 1 Female ☐ 2 Diverse ☐ 3

11) German citizenship: Yes ☐ 1 No ☐ 2

12) Focus of work: Science ☐ 1 Administration/Infrastructure ☐ 2

13) What is your highest general school-leaving qualification?

- ☐ 1 Secondary school
- ☐ 2 Secondary school leaving certificate
- ☐ 3 A-levels
- ☐ 4 No degree

14) Space for remarks:

©2023 This work is licensed under Creative Commons Attribution 4.0 International
